# Editorial: Health literacy and disease prevention

**DOI:** 10.3389/fpubh.2023.1128257

**Published:** 2023-03-28

**Authors:** Deep Shikha, Poonam Kushwaha, Ozden Gokdemir, Roy Rillera Marzo, Sudip Bhattacharya

**Affiliations:** ^1^Department of Community Medicine, Himalayan Institute of Medical Sciences, Swami Rama Himalayan University, Dehradun, India; ^2^Department of Community Medicine, Rama Medical College Hospital and Research Centre, Kanpur, Uttar Pradesh, India; ^3^Faculty of Medicine, Izmir University of Economics, Izmir, Türkiye; ^4^Department of Public Health, Management and Science University, Shah Alam, Selangor, Malaysia; ^5^Department of Community and Family Medicine, All India Institute of Medical Sciences, Deoghar, India

**Keywords:** health literacy, disease prevention, health promotion (HP), disease control, global health

## Introduction

Health literacy (HL) is the degree to which individuals have the capacity to obtain, process, and understand the basic health information and services needed to make appropriate health decisions accordingly (1). It is also defined as a collection of skills that includes reading, listening, analysis, decision-making, and the ability to apply these skills to health problems; it is not necessarily tied to years of school or general reading proficiency (2).

Health literacy concerns “the knowledge and competencies of persons to meet the complex health demands in modern society.” Hence, an integrative conceptual model of HL containing 12 dimensions, referring to “the knowledge, motivation and competencies of accessing, understanding, appraising and applying health-related information within the healthcare, disease prevention and health promotion setting,” has been developed by Sørensen et al. (3).

HL focuses beyond behavior-oriented communication and the concept of health education. It encompasses various determinants of health, including the environmental, organizational, social, and political. Environmental health literacy *per se* is a relatively new framework to conceptualize “how people understand and use information about potentially harmful environmental exposures and their influence over health” (4). Organizational health literacy, which comprises six main categories (25 subcategories), aims to respond to the HL needs of patients by improving health information and services and making them easier to understand, access, and apply (5).

Health literacy implies the achievement of a level of knowledge, skills, and confidence to take action to improve personal and community health by changing personal lifestyles and living conditions. In addition, by improving people's access to health information and their capacity to use it effectively, health literacy is critical to empowerment (6).

The Frontiers in Public Health e-book on Health Literacy and Disease Prevention, volume I, includes 26 research articles with over 154 authors and many more reviewers from more than 15 countries. This volume highlights the need to contribute to future research perspectives, culminating knowledge, policy-making, and their implementation in the field of health literacy and disease prevention.

## Role of health literacy in maternal health

The need for health literacy and health promotion is also important for attaining positive maternal health outcomes. This is more challenging in low- and middle-income countries, which are facing scarcity of resources and lack of awareness about existing policies.

The opinion paper by Shahil Feroz emphasized the strengthening of self-care agency to improve self-care throughout pregnancy for better maternal outcomes, including the training of healthcare staff, community health workers, adopting digital health technologies to perform self-care at home, and coaching clinicians to identify areas where their self-care agency is compromised. It also focused on increasing resources to penetrate cell phones among pregnant women to empower and support them, thereby increasing their self-care agency. Telemonitoring use, motivation, decision-making skills, and awareness of pregnancy are some measures that can help health workers to intervene in good time and appropriately for the prevention of diseases in pregnancy and complication readiness.

## Health promotion in preventing chronic diseases

HL, as a crucial tool in education and communication for the prevention of non-communicable diseases, ought to comprise sustainable, long-term measures that start early in the life course (7).

Dietary habits and lifestyle modifications play a very crucial role to either curb or double or triple the burden of diseases, with rising trends in chronic lifestyle and non-communicable diseases (NCD). The importance of front-of-package food labels (FOPL) has been recognized by many public health authorities for helping the consumer to make healthy choices while selecting for day-to-day food items. Bhattacharya et al. conducted a country-wide (2,024 respondents) online survey on “consumer's perception on FOPL in India.” The authors concluded that many consumers are aware of the nutrition labels and consider them while making food purchases. They also supported the idea of placing the food labels on the front of packets. The need for more evidence-based research, policy formation, and implementation was emphasized. The study gave a key message: “*what to eat and how to select*.”

Kozhakhmetova et al. emphasized that a low level of awareness among physicians in Kazakhstan about celiac disease (CD) could lead to the incorrect use of diagnostic tests, delay in diagnosis, and inefficient treatment. Around 232 representatives participated in this online study, and knowledge about the disease etiology (autoimmune origin) was demonstrated by about one-fourth of the participants. The total scores revealed that more than half of primary care physicians had poor knowledge of CD, but the majority intended to learn more about the causes, diagnosis, and treatment. They also revealed many myths among healthcare professionals regarding CD.

Chronic diseases pose a great burden on any country's economy and a physical, social, and mental setback for the patient and their family. Mu et al. reported that hypertensive people in China had a low level of diet-related knowledge, attitudes, and behaviors. Therefore, it is necessary to strengthen dietary education and interventions directed at high-risk groups, especially in resource-poor settings. Such interventions can be beneficial to delay or mitigate the onset of disease, complications, and disability limitations.

Karasiewicz et al. in accordance with the European Code Against Cancer (ECAC), tried to propose a fresh approach for locating and addressing any shortcomings in health education services. They concluded that PHC has an integral role in promoting cancer prevention knowledge, along with the involvement of health professionals to promote the ECAC. More resources need to be mobilized to support PHC staff and decision-makers for local health promotion needs.

Xu et al. worked to improve cardiac emergency preparedness by creating a team-based CPR educational plan. It includes a three-person team and emphasizes task allocation, leadership, and closed-loop communication. This plan proved to be very feasible for improving bystanders' teamwork performance and effective for improving resuscitation quality in pre-arrival care. Wide application of this team-based CPR plan in the educational program for better resuscitation performance in real rescue events can improve emergency preparedness.

van der Gaag et al. explored the preferences regarding the self-management outcomes of chronically ill patients with limited health literacy and their association with sex, comorbidity, and age. The outcome was satisfaction with care, including “overall satisfaction,” “communication with health care providers,” and “provision of information and trust.” The study emphasized that there is need to explore the discussion between healthcare professionals and patients about the variety of outcomes for patients' self-management.

Azkan Ture et al. emphasized the role of health literacy in chronic obstructive pulmonary disease (COPD) patients in Turkey. There was a significantly inverse proportional association of HL with the severity of COPD. Thus, while assessing such patients, healthcare professionals should be watchful in their interpretation, understanding, and should help those adopting preventive measures in view of the low health literacy among them.

## Health literacy in COVID-19

When COVID-19 evolved rapidly, health literacy emerged as an important tool for its prevention. Though it was difficult to enhance HL during the pandemic, immediate action was required for individual and system preparedness to solve complex real-life problems (8).

According to a health belief model in China assessed by Zhang et al., the relationship with HL affected health behaviors in the context of COVID-19. The findings support the idea that HL plays a critical role in the adoption of preventive health behaviors such as lifestyle behaviors (e.g., exercise, alcohol consumption, medical check-ups, and smoking) and preventive behaviors related to COVID-19 (e.g., wearing masks, hand washing, crowd avoidance, and taking the COVID-19 vaccination). HL has a direct effect on health behaviors and its three constructs (perceived susceptibility, perceived severity, and perceived barriers) in the health belief model, as well as an indirect effect on health behaviors *via* the increasing perceived barriers related with COVID-19 preventive measures. The authors provided a theoretical basis for stakeholders to develop effective policies and interventions and for promoting people's initiative and self-discipline in forming a healthy lifestyle.

Rezakhani Moghaddam et al. recruited 380 people in Iran between 18 and 65 years of age through cluster sampling to determine the role of e-health literacy (e-HL), self-efficacy, sociodemographic factors, and some cognitive factors in adopting protective behaviors against COVID-19. They found a significant association of gender, occupation, education, income, and marriage with e-HL and COVID-19 preventive behaviors. Perceived self-efficacy was the strongest factor in COVID-19 protective behaviors in adults. The study addressed the main factors involved in adopting protective, promotive, and preventive behaviors during the COVID-19 pandemic. This could be helpful for policy-making and to perform interventions (designing and using health messages and e-platforms) to improve e-HL and self-efficacy with a special focus on low socioeconomic status (SES) groups.

Baba et al. from Democratic Republic of Congo, highlighted the importance of engaging faith communities and leaders during health emergencies to spread health messaging. They described that capacity building, training, and working with health officials could help them to create more effective health messaging.

Chen et al. conducted a study among 5,596 residents aged 15–69 in Zhejiang, China, using multistage, stratified probability proportional to the size sampling. The study reported that residents with adequate HL were more likely to have better COVID-19 awareness. The three dimensions of HL (knowledge and attitudes, behavior and lifestyle, and health-related skills) were found to be associated with COVID-19 awareness. Additionally, COVID-19 awareness was associated with age, occupation, family size, annual household income, and chronic conditions. They recommended that promotion of HL should be performed in response to the present COVID-19 pandemic and possible public health emergencies in the future as well.

New “normal” guidelines and a series of “mobile health applications” have been introduced and implemented to aid disease control monitoring and prevention of widespread COVID-19 outbreaks in Thailand. The knowledge, attitudes, and practices (KAP) among the Thai people regarding these guidelines and quality of life (QOL) were assessed by Waewwab et al.. Accurate knowledge of COVID-19 was associated with higher education, being a government employee, monthly family income > 30,000 Thai Baht, and regular use of social media. More positive attitudes were associated with positive practices. The author recommended that more work should be done to implement mobile health applications and to improve their ease of use and promotion strategies.

Lee et al. assessed the knowledge, perception, and behaviors of the staff and students of Universiti Tunku Abdul Rahman with respect to COVID-19 amidst the third wave of the infections. They were not able to establish a statistical difference in the total score of knowledge on COVID-19, self-risk perception, preparedness and perceived self-efficacy, preventive (own) measures, or unwanted and desirable behaviors between staffs and students. The perceived self-risk was relatively low, implying that the respondents considered themselves to be quite safe. Therefore, despite the introduction of the vaccination, frequent and timely updates of information and reminders *via* social media, television, and radio regarding the deadly nature of the virus became crucial to support the efforts to battle COVID-19.

Dam et al. from Austria, underlined that a structured questionnaire may be a less efficient tool to measure knowledge transfer in health-related topics among people with intellectual disabilities than conducting semi-structured interviews. Furthermore, the results of this study showed the impact and usefulness of images on the comprehension of the subject matter. Potential barriers in knowledge acquisition and application among people with intellectual disabilities were pointed out. The importance of developing inclusive strategies to improve health-related knowledge transfer to enhance HL and related outcomes was highlighted too.

Schools are considered to be fundamental centers and an effective instrument for developing community health-related behaviors. Deressa et al. conducted a study on the involvement of primary school students in promoting preventive actions against malaria both in schools and communities. They evaluated the perceived outcomes of peer learning and the educational approach on malaria prevention (PLEA-Malaria) in rural communities in Ethiopia. Moreover, peer educators thought the program was well-implemented. Therefore, it was advised that while developing and executing such programs in schools, a person's self-efficacy, risk perceptions, and peer education team spirit should be considered.

## Perspectives

This Research Topic not only emphasized the importance of HL and disease (communicable and non-communicable) prevention but also highlighted the various dimensions of HL and appropriate health-related behavior to combat present and future public health problems.

## Author contributions

All the authors have made a substantial, direct, and intellectual contribution to the work and approved it for publication.
